# Effect of *Astragalus membranaceus* extract on diabetic nephropathy

**DOI:** 10.1530/EDM-14-0063

**Published:** 2014-09-01

**Authors:** Jiman Kim, Eulsun Moon, Seungwon Kwon

**Affiliations:** 1 Kyunghee-saeng Korean Medicine Clinic, Seoul, Korea; 2 Seoul 365 Medical Clinic, Seoul, Korea; 3 Department of Cardiovascular and Neurologic Diseases, College of Korean Medicine, KyungHee University, 1 Hoegi-dong, Dongdaemun-gu, Seoul, 130-702, Korea

## Abstract

Diabetic nephropathy, a microvascular complication of diabetes, is a progressive kidney disease caused by angiopathy of the capillaries in the kidney glomeruli. Herein, we report a case of a 62-year-old patient with a 30 year history of diabetes, who showed a substantial improvement in diabetic nephropathy on administration of 30 g of *Astragalus membranaceus* extract per day. After 1 month, estimated glomerular filtration rate increased from 47 to 72 ml/min per 1.73 m^2^ and was subsequently maintained at the 1-month follow-up. Urinary protein levels also decreased following treatment. Herein, we present and discuss the evidence and mechanism of *A. membranaceus* on diabetic nephropathy in this patient.

**Learning points:**

- Diabetic nephropathy is a progressive kidney disease.
- Angiotensin-converting enzyme (ACE) inhibitors or angiotensin receptor blockers (ARBs) are currently used to prevent and delay the progression of diabetic nephropathy. However, their effects are not sufficient to prevent a decline in kidney function.
- Furthermore, combination therapy with an ACE inhibitor and an ARB can produce adverse effects without additional benefits.
- In the early phase of diabetic nephropathy, administration of *Astragalus membranaceus* can be a therapeutic option.

## Background

Diabetic nephropathy, a microvascular complication of diabetes, is a progressive kidney disease caused by angiopathy of the capillaries in the kidney glomeruli. The principal cause of diabetic nephropathy, a prime indicator for dialysis, is long-standing hypertensive diabetes mellitus, characterized pathophysiologically by glomerular hyperfiltration. Angiotensin-converting enzyme (ACE) inhibitors and angiotensin receptor blockers (ARBs), both commonly used in preventing diabetic nephropathy, act on this pathophysiology (1). However, the preventative and therapeutic effect of combined ACE inhibition and ARB therapy on diabetic nephropathy is still insufficient to maintain kidney function (2). In addition, the combined use of ACE inhibitors with ARBs offers no discernible benefit and is associated with an increase in adverse effects (3). In this case study, we present a patient with established diabetes showing a dramatic decrease in proteinuria and increase in estimated glomerular filtration rate (eGFR) with substantial improvement in diabetic nephropathy on administration of *Astragalus membranaceus* extract. Furthermore, we discuss the evidence and mechanism of effect of *A. membranaceus* on the pathophysiology of diabetic nephropathy.

## Case presentation

A 62-year-old man (weight, 68 kg; height, 170 cm; waist, 86.36 cm) with controlled diabetes mellitus, hypertension, dyslipidemia, diabetic retinopathy, diabetic foot (foot ulcer), and early diabetic nephropathy presented at Kyunghee-saeng Korean Medicine Clinic. He was previously diagnosed with type 2 diabetes at another hospital and had been receiving oral hypoglycemic agents to control blood glucose levels from 1984 until 2000 when he suffered a stroke. His blood glucose levels were subsequently found to be poorly controlled and therefore insulin injection therapy was recommended. At the same time, diabetic retinopathy was also detected, which was treated by a combination of laser photocoagulation and an adjusted calcium dobesilate dose of 250 mg/day. The patient subsequently modified his lifestyle and continued his medication. In 2011, amputation of the right second and third toes was performed due to the presence of diabetic foot ulcers. Insulin therapy was discontinued in January 2012 because of several hypoglycemic events and oral hypoglycemic therapy was resumed (sitagliptin 100 mg/day, metformin 500 mg/day). However, in November 2013, diabetic nephropathy corresponding to stage 3 chronic kidney disease (CKD) was detected by routine medical checkups and treatment with Perindopril commenced. This failed to improve kidney function and he presented at our clinic on April 7, 2014, seeking to recover his renal function level with herbal medicine.

## Investigation

At presentation, fasting levels of glucose and HbA1c were 4.884 mmol/l (88 mg/dl) and 42 mmol/mol (6.0%), respectively, showing adequate blood glucose control. The eGFR was 47 ml/min per 1.73 m^2^ as examined by the Modification of Diet in Renal Disease (MDRD) equation, indicating stage 3 CKD. This corresponded with a serum creatinine level of 122.0 μmol/l (1.6 mg/dl). The urinary protein levels were 53 mg/dl.

## Treatment

The patient continued to receive their existing medications: hypoglycemic agents (sitagliptin 100 mg/day, metformin 500 mg/day), antilipidemic agent (atorvastatin 10 mg/day), antihypertensive agents (diltiazem 180 mg/day, perindopril 16 mg/day), antiplatelet agents (aspirin 100 mg/day, clopidogrel 75 mg/day), *Ginkgo biloba* extract 160 mg/day, and calcium dobesilate 750 mg/day. In addition, the patient took *A. membranaceus* extract (30 g/day) three times a day until June 7, 2014.

## Outcome and follow-up

**Figure 1:**
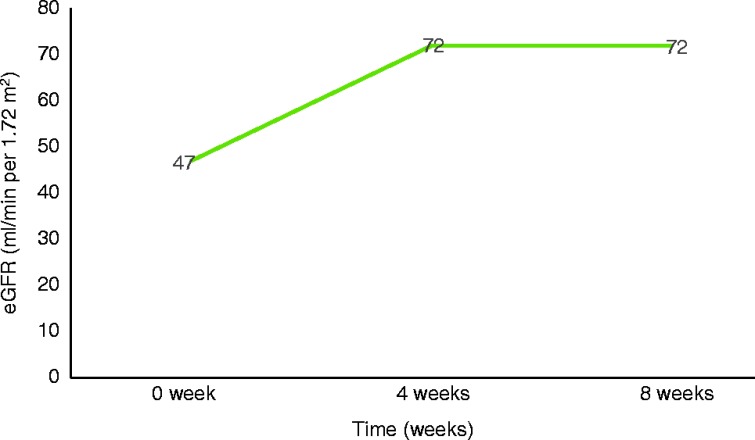
Changes in estimated glomerular filtration rate (eGFR). 0 week: April 7, 2014; 4 weeks: May 3, 2014; 8 weeks: June 7, 2014.

**Figure 2:**
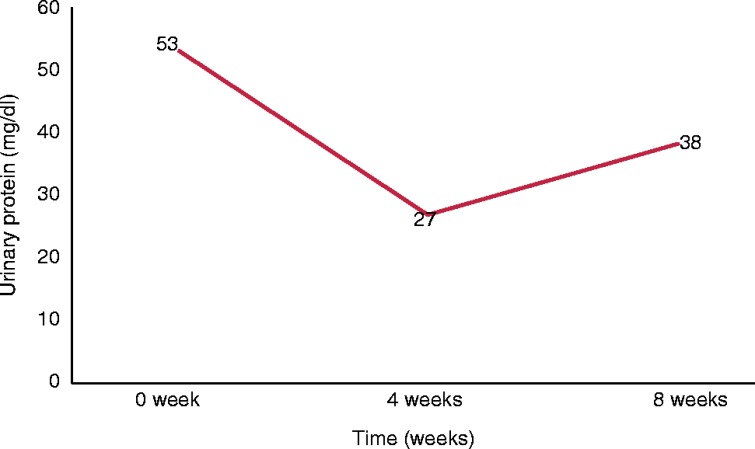
Changes in urinary protein. X axis, time in weeks; Y axis, urinary protein (mg/dl); 0 week, April 7, 2014; 4 weeks, May 3, 2014; 8 weeks, June 7, 2014.

During the administration of *A. membranaceus* extract, regular tests were performed once a month to check for its effect on diabetic nephropathy. After 1 month, eGFR increased from 47 to 72 ml/min per 1.73 m^2^, which was maintained at the 1-month follow-up (Fig. 1). After 1 month, urinary protein levels decreased from 53 to 27 mg/dl but were increased slightly to 38 mg/dl at follow-up (Fig. 2).

Other parameters such as, glucose level, HbA1c and cholesterol were within the normal range during the follow-up period.

## Discussion

We demonstrate an improvement in eGFR in a patient with established diabetic nephropathy after treatment with *A. membranaceus* extract for 2 months. With *A. membranaceus* extract administration, kidney function improved from stage 3 CKD, which is indicative of chronic renal failure, to stage 2, thus reversing renal failure. Furthermore, urinary protein output also decreased compared with initial levels.

Diagnosis of diabetic nephropathy is based on the estimation of urinary albumin or eGFR by the Cockroft-Gault or MDRD equation. Stages of CKD as defined by eGFR are from stage 1 to 5 (stage 1: ≥90; stage 2: 60–89; stage 3: 30–59; stage 4: 15–29, and stage 5: <15 ml/min per 1.73 m^2^ respectively). In this case, the patient's eGFR corresponded to stage 3 CKD which was indicative of chronic renal failure. Despite 5 months of ACE inhibitor administration, controlled blood pressure, and controlled blood glucose level, there was no improvement in disease status. Therefore, the patient gave his consent to determine the effect of herbal medication to improve his condition.

We chose *A. membranaceus* extract based on the following reasons. A meta-analysis (4) showed that *A. membranaceus* injection had a more therapeutic effect, including renal protective effect and systemic state improvement, in diabetic nephropathy patients than in control patients in the clinical situation. Another meta-analysis (5) focusing on animal models suggested that *A. membranaceus* is effective in reducing fasting blood glucose and albuminuria levels, reversing glomerular hyperfiltration and ameliorating the pathological changes in early diabetic nephropathy models. An experimental study (6) (7) suggested that the mechanisms of *A. membranaceus* action in diabetic nephropathy are a reduction in the mRNA level of nuclear factor kappa B (*NF* *κ* *B*), which has been suggested to play a key role in the pathogenesis of diabetic nephropathy, and an increase in inhibitory NFκB protein mRNA expression in the renal cortex. Inhibition of high glucose-induced early mesangial cell proliferation and advanced glycation end-product (AGE)-induced endothelial cell apoptosis via effects of calycosin and calycosin-7-*O*-β-d-glucoside, two major isoflavonoids present in *A. membranaceus*, are also suggested to contribute to the beneficial effect of *A. membranaceus* on diabetic nephropathy (8).

Diabetic nephropathy is the single most common cause of end-stage renal disease (ESRD) (9). It is a progressive disease involving multiple factors including metabolic and hemodynamic alterations, oxidative stress, activation of the renin–angiotensin system, and inflammation (release of cell adhesion molecules, growth factors, chemokines, and pro-inflammatory cytokines) (10). In the present case, there was an improvement in diabetic nephropathy following administration of *A. membranaceus* for 2 months, although from this data we cannot draw conclusion of which pathway was affected.

Because of the short follow-up period, we also cannot be certain of the long-term effect of *A. membranaceus* administration on this patient's progressive nephropathy. However, this case provides compelling evidence to support the administration of *A. membranaceus* as a treatment to prevent the progression of diabetic nephropathy and improve kidney function, especially in the early stages of the disease.

## Patient consent

Written informed consent has been obtained from the patient for publication of the submitted article.

## Author contribution statement

Jiman Kim treated this patient. Seungwon Kwon wrote this manuscript and reviewed this case. Eulsun Moon was involved in critical revision of the manuscript.
